# Editorial: Perturbation of RNA Binding Protein Regulation in Cancer

**DOI:** 10.3389/fgene.2021.693766

**Published:** 2021-06-24

**Authors:** Yongsheng Li, Juan Xu

**Affiliations:** ^1^Key Laboratory of Tropical Translational Medicine of Ministry of Education, College of Biomedical Information and Engineering, Hainan Medical University, Haikou, China; ^2^College of Bioinformatics Science and Technology, Harbin Medical University, Harbin, China

**Keywords:** RNA binding protein, cancer, network, method, survival

RNA-bind proteins (RBPs) are typically types of proteins that bind RNA to play critical roles in development or cancer (Pereira et al., 2017; Mohibi et al., 2019). Recent studies have identified thousands of RBPs and also revealed the dysregulation of RBPs in various kinds of cancer types, such as mutation (Chen et al., 2019), copy number variation (Xu et al., 2019), expression perturbation as well as perturbations of RBP-gene regulation (Zhang et al., 2020).

With the development of high throughput sequencing technology, some recent studies have highlighted precise dysregulated RBPs in specific cancers. Lung cancer is the leading cause of deaths worldwide and dysregulation of RBPs has been found in lung squamous cell carcinoma (LUSC). Li et al. analyzed the gene expression and clinical information from The Cancer Genome Atlas (TCGA) and observed 300 aberrantly expressed RBPs. These RBPs were mainly associated with mRNA metabolic processes, RNA modification and cancer-related signaling pathways. Moreover, they identified nine RBP genes for constructing a prognostic model in LUSC. In another study, Zhang et al. characterized the clinical relevance of RBPs in colorectal cancer. First, 242 differentially expressed RBPs were identified and eight RBPs were found to be related with the prognoses of colorectal cancer patients. Four RBPs (NOL3, PTRH1, UPF3B, and SMAD6) were used to construct the prognostic risk score model. In addition, Zhong et al. also constructed a prognostic model based on RBP expression in kidney renal clear cell carcinoma. Furthermore, potential drugs for cancer were predicted based on the Connectivity Map database. Moreover, RBPs were also play important roles during cancer progression (Wang et al.).

In addition, although some targets of RBPs were identified based on computational or experimental methods, the genome-wide RBP-gene regulatory network in cancer is largely unknown and little is known about the synergetic interaction between RBPs and other regulators. In recent studies, co-expression network analysis was applied to predict the function of RBPs (Wu et al.). In the past decade, these studies about RBPs mainly focused on mutations in RBPs or their target genes. However, it has been increasing appreciated that many driver mutations might perturb molecular interactions or regulatory networks (Mosca et al., 2015; Yi et al., 2017). Recently, a computational method Mutational Effect on RNA Interactome Topology (MERIT) was proposed to analyze the RBP-gene regulatory networks across cancer types (Li et al., 2019a). All these results provide insights into characterizing perturbed RBP-RNA regulatory networks in cancer, as well as the genotype-phenotype relationships underlying human cancers, and RBPs are potential biomarkers for precision medicine.

The methylation of N6 adenosine (m6A) plays a critical role in diverse biological processes (Li et al., 2019b). Moreover, recent studies have revealed that RBPs also play important roles in RNA methylation. IGF2BP3 was identified as a potential oncogene across multiple cancer types and also play important roles in tissue development (Xu et al., 2021; Zhang et al., 2021). These studies provide another regulatory layer of RBPs in cancer.

In summary, RBPs play important roles in cancer development and progression. All these integrated analysis provided detailed knowledge of the function of the RBPs in cancer, which will facilitate the development of rational therapies for cancer.

## Author Contributions

All authors listed have made a substantial, direct and intellectual contribution to the work, and approved it for publication.

## Conflict of Interest

The authors declare that the research was conducted in the absence of any commercial or financial relationships that could be construed as a potential conflict of interest.
